# A Review of Mitral Isthmus Ablation

**DOI:** 10.1016/s0972-6292(16)30522-8

**Published:** 2012-07-28

**Authors:** Kelvin CK Wong, Timothy R Betts

**Affiliations:** Oxford Heart Centre, Oxford University Hospitals NHS Trust, Oxford, United Kingdom

**Keywords:** mitral isthmus, atrial fibrillation, ablation

## Abstract

Mitral isthmus ablation forms part of the electrophysiologist’s armoury in the catheter ablation treatment of atrial fibrillation. It is well recognised however, that mitral isthmus ablation is technically challenging and incomplete ablation may be pro-arrhythmic, leading some to question its role. This article first reviews the evidence for the use of adjunctive mitral isthmus ablation and its association with the development of macroreentrant perimitral flutter. It then describes the practical techniques of mitral isthmus ablation, with particular emphasis on the assessment of bi-directional mitral isthmus block. The anatomy of the mitral isthmus is also discussed in order to understand the possible obstacles to successful ablation. Finally, novel techniques which may facilitate mitral isthmus ablation are reviewed.

## Background

Based on the multiple wavelet hypothesis, the Cox-maze procedure was designed using linear lesions to compartmentalize the atria and to interrupt potential macroreentrant circuits which may maintain atrial fibrillation (AF) [1]. Interventional electrophysiologists have subsequently emulated the surgical technique by attempting to perform radiofrequency linear ablation with varying degrees of success. Creating contiguous and transmural linear lesions using the "point-by-point" technique has proven to be challenging. Of the several proposed linear lesions, only mitral isthmus and roof line ablation remain commonly performed as an adjunct to pulmonary vein isolation in the treatment of atrial fibrillation [2,3].

A left atrial "isthmus" was first described by Luria et al [4]. They described the phenomenon of intra-atrial conduction block in a subset of patients undergoing ablation for left lateral accessory pathways. This conduction block was manifested by changes in local ventriculo-atrial time at coronary sinus (CS) recording sites and a change in the retrograde atrial activation sequence. They hypothesized that this conduction block was due to inadvertent damage to a narrow "isthmus" of myocardium between the lateral mitral annulus and the left inferior pulmonary vein (PV), analogous to the cavotricuspid isthmus of the right atrium. Due to the ease of achieving inadvertent block at the isthmus, they further suggested that this left atrial "isthmus" was an attractive target for treatment of atypical flutter which utilised the isthmus for the macroreentrant circuit and also for left atrial compartmentalization procedures in the treatment of atrial fibrillation. While ablation at this left atrial isthmus has been reported in isolated case reports for the treatment of atypical perimitral flutter, its feasibility and safety in AF ablation was unreported until described by Jais et al. in 2004 [3].

An ideal linear lesion should connect adjacent anatomic structures or areas of scar which are barriers to electrical impulse propagation, should be as short as possible to avoid the risk of "gaps", and should be easy to perform with a low risk of complications. Furthermore, it should be amenable to easy testing for bidirectional block along the line, allowing for a clear endpoint. In many respects, the mitral isthmus initially appeared to fulfil these requirements: It is relatively well defined and anatomically bounded by the mitral annulus and left inferior PV ostium (or the anterior/inferior border of ablation lesions during circumferential ablation of the left sided pulmonary veins) and its position at the lateral mitral annulus adjacent to the left atrial appendage (LAA) and the coronary sinus (CS) facilitates assessment for bi-directional block. However, while mitral isthmus block was initially observed with limited ablation for left lateral accessory pathway, it is now well recognised that mitral isthmus ablation is challenging and may be associated with significant complications (see later).

## Mechanism

While the pathophysiology of AF is still not fully understood, a commonly accepted model is that AF may be initiated by triggers or drivers which arise commonly but not exclusively from the pulmonary veins, and is maintained or perpetuated by a suitable substrate, under the modulation of the autonomic nervous system. While it is also not always clear how mitral isthmus ablation may contribute to the treatment and prevention of atrial fibrillation, the therapeutic efficacy of mitral isthmus ablation is likely to be mediated through its impact on substrate modification, elimination of triggers, prevention of macroreentry around the mitral annulus or left PVs, modification of cardiac autonomic innervation and atrial de-bulking.

It is likely that mitral isthmus ablation modifies a large region of the LA substrate for AF by eliminating anatomic or functional reentry involving both the mitral isthmus and left-sided pulmonary veins [3]. This is evidenced by the transformation from atrial fibrillation to atypical macroreentrant perimitral flutter either during the index procedure of AF ablation or follow up, particularly when ablation is performed in a step-wise approach [5-6]. Its elimination by mitral isthmus ablation appears to be an obligatory step on the way to sinus rhythm in a significant subset of patients. In addition to its possible role in the interruption of perimitral flutter, mitral isthmus ablation may achieve substrate modification by compartmentalisation and reduction of critical mass needed for fibrillatory conduction, especially when combined with other linear ablations.

Due to the limitations of Moe's multiple wavelet hypothesis [7], there have been suggestions that a number of focal high frequency sources may drive and perpetuate AF [8]. These "mother rotors" may give rise to "vortices of electrical activation" (spiral waves) which are self-sustaining and may be stationary or may drift and subsequently anchor to anatomical heterogeneities in the cardiac muscle, i.e. the PV-LA junctions. Mitral isthmus ablation may target these "rotors". The cardiac autonomic nervous system, with vagal activation, in particular has been implicated in the pathogenesis of atrial fibrillation. In human studies, adjunctive modification of the autonomic tone with pulmonary antral ablation has been demonstrated to be associated with favourable results [9]. Armour et al. observed ganglionated plexi consistently located in 5 atrial regions [10]. One such region is the infero-lateral aspect of the posterior LA, which may be targeted by ablation at the mitral isthmus area.

Mitral isthmus ablation may eliminate arrhythmogenic triggers arising from the ligament of Marshall, an epicardial structure that has been proposed as a source of non-pulmonary ectopy which may trigger AF [10]. The ligament of Marshall (LOM) was first described in 1850 as a "vestigial fold of the pericardium" and is the remnant of the embryonic left superior vena cava. It is located on the epicardial aspect of the left lateral ridge which lies in between the left atrial (LA) appendage and left pulmonary veins (PVs). It consists of blood vessels (the oblique vein of Marshall and arterial branches), myocardial sleeve (Marshall bundle) and autonomic nerves. The Marshall bundle has variable connections to the CS musculature and the LA myocardium in the region of the left lateral ridge. Studies have elegantly demonstrated that the Marshall bundle is both an electrical and an autonomic nervous conduit, possessing properties which may implicate it in the pathogenesis of AF [12,13]. It has also been shown to be a source of ectopy which may trigger AF [14]. Recordings from the LOM during persistent AF have shown rapid activation and complex fractionated atrial electrograms which may be associated with driving or perpetuating atrial fibrillation [15,16]. Ablation of LOM resulted in vagal denervation and blunting of vagally-mediated reduction of atrial tissue effective refractory period which may reduce the vulnerability to AF [13]. More recently, LOM is shown to be a critical component of the perimitral circuit and ethanol ablation has been shown to be feasible and facilitates the achievement of bi-directional mitral isthmus block [17].

## Clinical efficacy/literature review

AF cycle length is a surrogate measure of local atrial refractoriness and has been shown to prolong progressively or immediately prior to termination by drugs [18]. It has been suggested as a useful marker of substrate changes during catheter ablation for persistent AF, with prolongation indicating participation of the targeted tissue in the AF process. Experimental studies employing linear lesions have shown that they produce prolongation of the AFCL without changing conduction velocity or the number of wavelets. In human studies, 40% of patients had significant AFCL prolongation during linear ablation [5,19]. Specifically, mitral isthmus ablation was found to increase the AFCL by about 20 ms which is comparable to that observed during pulmonary vein isolation [20]. Mitral isthmus blation was also associated with the conversion of AF to sinus rhythm or atrial tachycardia, thus further supporting its role in substrate modification. In fact, linear ablation (mainly mitral isthmus) accounted for about 20% of cases with AF termination.

Using the Bordeaux "step wise" approach, AF often converts to atrial tachycardia or flutter and elimination of the flutter by linear ablation appears to be an obligatory step on the way to sinus rhythm. This may happen during the index procedure or during redo-ablations. In fact, Knecht et al. reported that 86% of 180 patients undergoing catheter ablation for persistent AF eventually had mitral isthmus ablation. Patients who did not have both roof and mitral isthmus ablation during the index procedure were found to have a significantly higher incidence of macroreentrant flutter during follow up [21].

Mitral isthmus ablation also resulted in significantly reduced atrial frequency as measured from surface 12 lead ECG recordings using a combination of principal component and Fourier transform algorithms [22]. More recently, mitral isthmus ablation was demonstrated to eliminate spectral components (lower frequency than the dominant frequency) when bi-directional block was achieved even though they were not affected by pulmonary antral ablation and ablation of complex fractionated atrial electrograms [23]. This led the authors to speculate that these spectral components represent macroreentrant flutters which may co-exist with AF and act as lower frequency drivers of AF.

Several studies had reported on the use of linear lesions as a treatment strategy for the catheter ablation of AF. Most centres have used it as an adjunct to pulmonary vein isolation while others have reported a purely linear approach, i.e the "figure-7 lesion line". However, there is limited data on the true clinical efficacy of mitral isthmus ablation.

The clinical studies which assessed the efficacy of added mitral isthmus ablation (either alone or in combination with roof line) are summarised in Table 1. 6 of the 8 studies showed a significant incremental benefit of additional mitral isthmus ablation [3,24-28]. The magnitude of the benefit appeared to be larger for persistent AF (20-40%) compared to paroxysmal AF (15-20%). This benefit was both in the reduction of atrial fibrillation and of macroreentrant flutter. One short term study (only 6 months follow up) showed a reduced success with mitral isthmus ablation combined with circumferential pulmonary vein ablation [29].

## Mitral isthmus ablation, resumption of conduction and perimitral flutter

It is clear from many studies that complete lines lead to better outcomes [21,24,25,30]. Incomplete lines may be proarrhythmic. Fassini et al. showed that patients who had additional mitral isthmus ablation but did not achieve bi-directional block did not fare better than patients who had pulmonary vein isolation only [24]. Willems et al. also reported that the majority of patients who had recurrence from their substrate modification group had at least one incomplete linear lesion [25]. In his randomised trial, Pappone et al. found that additional linear ablation (roof and mitral isthmus) significantly reduced the incidence of macroreentrant flutters without affecting AF recurrence [27]. In a group of patients with persistent AF treated with ablation using the Bordeaux stepwise approach, Knecht et al. found that patients who did not require linear ablations for AF termination experienced a significantly higher incidence of macroreentrant flutter (76%) either during the index procedure or follow up compared to patients who needed linear ablations for AF termination (35%). Incomplete mitral isthmus lines more than doubled the risk of developing perimitral flutter post-ablation [21].

Even when there is acute block, the phenomenon of resumption of conduction following radiofrequency ablation is well recognised and has previously been well characterised with cavotricuspid isthmus (CTI) ablation [31] and pulmonary vein isolation [32]. However, there is limited data on the recurrence of mitral isthmus conduction after an initially successful ablation. Based on a small number of patients having redo-procedures due to recurrence of AF and/or organised atrial tachycardia, the rate of resumption of conduction of the mitral isthmus in this selected population ranged from 62% to 90%, although there is clearly a selection bias [33-36]. In a prospective randomised study, Sawhney et al. reported that in patients with paroxysmal AF, mitral isthmus ablation increased the risk of subsequently developing atypical left atrial flutters [37]. More recently, the same group reported from a retrospective series a high incidence of mitral isthmus reconduction which was significantly associated with the development of mitral isthmus dependent flutter [36]. In another retrospective series looking at 50 patients who had perimitral flutters either during the index procedure or follow up, prior mitral isthmus ablation was associated with a higher risk of developing perimitral flutter, suggesting that mitral isthmus ablation may be pro-arrhythmic despite achieving bi-directional block acutely [38]. However, these findings are not reflected in the early prospective randomised trials listed in Table 2. More prospective randomised studies are needed to resolve this issue.

Rostock et al. found that the site of reconduction was predominantly located at the pulmonary venous end of the mitral isthmus line (66%) and rarely found epicardially in the CS (6%) [35]. This would fit with the peri-venous tissue having the greatest myocardial depth from a post-mortem study [39]. However, this is not reflected in other studies which reported a need for epicardial CS ablation in the majority of patients [36]. Our experience is that most reconnections occur at the annular end of the line which could be ablated either endocardially or epicardially. This may be explained by the "heat-sink" effect of the epicardial vessels located at the annular end of the line.

A gap in the mitral isthmus line is undoubtedly a pre-requisite for the development of post-procedure macroreentrant perimitral flutter. Nevertheless, it is clear from several studies that these "gaps" do not always lead to clinical tachycardia. In fact, the majority of these "gaps" do not manifest as perimitral flutter [35,36]. It may be because "gaps" alone are not sufficient to support macroreentrant tachycardia and other factors may be necessary for initiation and/or perpetuation of macroreentry. In fact, Bai et al. suggested that for treatment of perimitral flutter, the strategy of eliminating PV and non-PV triggers may be superior to mitral isthmus ablation [40].

## Acute success

Despite recent technological advancement and better understanding of the anatomy, mitral isthmus ablation remains technically challenging, often requiring substantial ablation (> 15 minutes of radiofrequency energy), high ablation powers (up to 50W) and epicardial ablation within the CS (about 70% of patients). In spite of this, acute success rates are only moderate (see Table 2).

## Assessment of block

An essential requirement for linear ablation is the creation of a contiguous and transmural line of lesions resulting in complete conduction block capable of preventing macroreentrant wavelets. Hence, the ability to assess for complete block is crucial. Validation of bi-directional conduction block has been previously described and is facilitated by its proximity to the distal CS (the great cardiac vein) and left atrial appendage (LAA) which are located on opposite sides of the mitral isthmus line.

**Figure 1:**
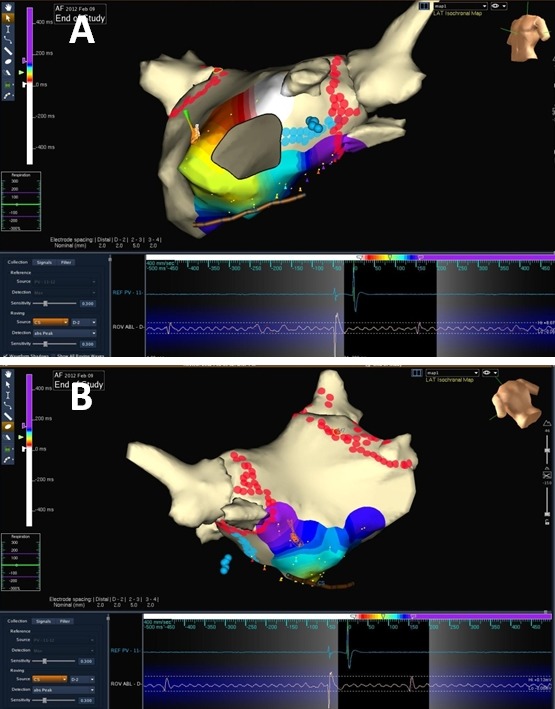
Left atrial 3D electroanatomical (EnSite Velocity, St. Jude Medical) activation maps (A: left anterior oblique; B: posterior) during left atrial appendage pacing in the presence of mitral isthmus block. There is counterclockwise activation around the mitral valve annulus resulting in a proximal to distal CS activation (white colour represents earliest activation and purple colour represents the latest activation). Red lesions represent circumferential pulmonary vein ablation and blue lesions represent mitral isthmus ablation.

**Figure 2:**
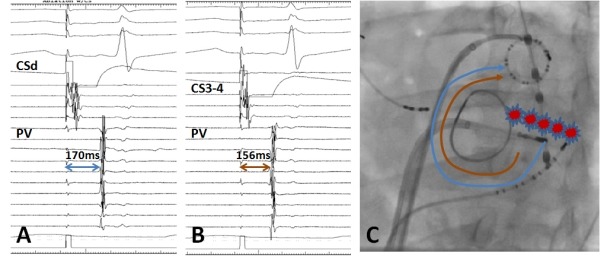
A: Electrograms during pacing from CS distal (CSd) bipole, showing time to the mulitpolar catheter (PV) positioned in left atrial appendage is 170ms. B: Electrograms during pacing from CS 3-4 bipole, showing time to the mulitpolar catheter (PV) positioned in left atrial appendage is 156ms. C: Left anterior oblique fluoroscopic image of a patient with previous mitral annular ring. Red lesions represent the mitral isthmus ablation. The blue and brown arrows indicate the distance travelled by the impulse when pacing at CSd and CS3-4 bipoles respectively when there is mitral isthmus block.

The general principles of assessing linear block apply and criteria for confirmation of bi-directional block across the mitral isthmus line are analogous to those used for assessing for cavotricuspid isthmus block: 1. Presence of widely spaced double potentials along the line when pacing is performed from either side of the line, i.e. "anteriorly" from the LAA or "posteriorly" from distal CS. 2. Activation mapping around the mitral valve annulus when pacing on either side of the line showing activation detours such that the activation time is latest adjacent to the line on the side opposite to the pacing site. When pacing "anterior" to the line (or from the LAA), there will be counter-clockwise activation around the mitral valve annulus and CS activation is proximal to distal (Figure 1). When pacing "posterior" to the line (i.e. distal CS), there is clockwise activation around the MVA. 3. Differential pacing utilizing 2 sites on one side of the line and measuring conduction time to a fixed point on the other side of the line. If there is complete block, then, pacing from the site closer to the line would result in a longer conduction time to the point on the other side of the line. E.g. conduction time from CS distal to LAA would be longer than from CS 3-4 to LAA (Figure 2)

Jais et al. described the validation of mitral isthmus block using only the ablation catheter and CS catheter [3]. The ablation catheter is moved along the line to check for widely spaced double potentials during distal CS pacing "posterior" (or "septal" in the paper) to the line. The ablation catheter can also be positioned just "anterior" to the line to check for differential pacing and confirmation of bi-directional block.

**Figure 3:**
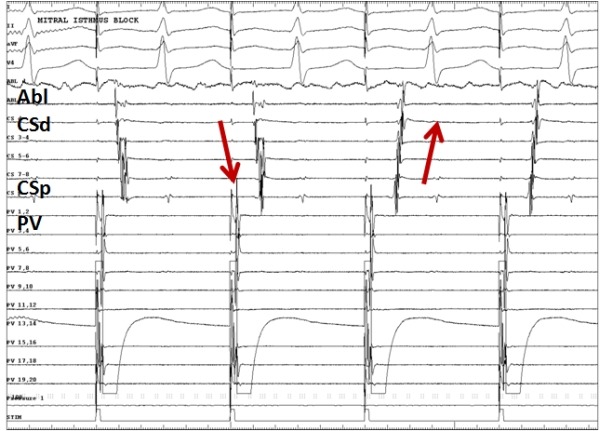
Electrograms showing the sudden change in CS activation from distal-to-proximal to proximal-to-distal (indicated by red arrows) when pacing from the multipolar catheter (PV) in the left atrial appendage, indicating mitral isthmus block. Abl indicates ablation catheter; CSd , coronary sinus distal bipole and CSp, coronary sinus proximal bipole.

At our institution, we prefer to perform mitral isthmus ablation during continuous pacing from the LAA (using the multipolar circular mapping catheter) as this allows for beat-to-beat assessment of mitral isthmus conduction to guide real-time ablation and enable the prompt recognition of mitral isthmus block (Figure 3). We have shown in a previous study that a change in CS activation from distal-proximal to proximal-distal predicts bi-directional mitral isthmus block with high specificity and sensitivity [41]. One of the pitfall of this technique is the appearance of "pseudoblock" (proximal to distal CS activation) secondary to the disconnection of the distal CS musculature from the atrial myocardium even when there may still be endocardial conduction across the mitral isthmus [42]. This can be unmasked by performing left atrial endocardial recording immediately posterior to the line to ensure timig is identical to or later than the distal coronary sinus electrograms and may be complimented by performing an activation map around the mitral valve annulus.

## Anatomical Characteristics and Mitral Isthmus Ablation

**Figure 4:**
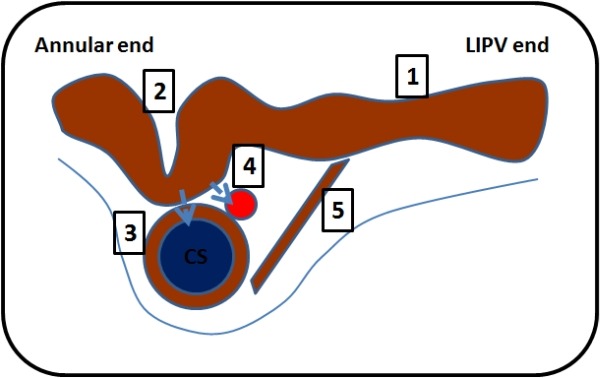
Schematic diagram of the cross section along the mitral isthmus from the mitral valve annulus to the left inferior pulmonary vein, showing the factors which may hinder mitral isthmus ablation. The blue vessel represents the coronary sinus (CS) and the red vessel represents the left circumflex artery. 1 indicates the long and thick mitral isthmus; 2, recess/pouch/crevice which may be difficult to get to; 3, muscular sleeve around the CS; 4, convective cooling by blood flow in the CS and artery ("heat-sink" hypothesis); 5, Epicardial connections (i.e. ligament of Marshall).

Previous post-mortem and in vivo studies using imaging modalities such as computed tomography, intra-cardiac ultrasound and fluoroscopic angiography have tried to elucidate the anatomy of the mitral isthmus to help guide catheter ablation and to identify obstacles to successful ablation [39,43-45]. It is clear from these studies that the anatomy of the mitral isthmus is far from uniform and exhibits significant variation. In post-mortem studies, it has an average length of about 35mm and depth of about 4mm. To put it into perspective, it is longer than the CTI with similar myocardial thickness. Certain anatomical features were suggested as possible obstacles to successful mitral isthmus ablation [39,43-45]: myocardial depth greater than 5 mm; convective cooling by local blood vessels such as the CS and circumflex artery; a myocardial sleeve around the CS and continuities with atrial myocardium which may bridge the lesion line; crevices in the isthmus area which may hinder safe and efficient radiofrequency energy delivery; continuation of atrial myocardium onto the atrial aspect of the mitral valve leaflet; and epicardial connections (e.g. the Ligament of Marshall) across the mitral isthmus line (Figure 4). Two studies have sought to identify the optimal position for the creation of the mitral isthmus line to avoid these anatomic obstacles, but it was clear that there was no fixed optimal position due to the anatomical heterogeneity in this area [43,44]. In general, the more proximal/"posterior" positions would be associated with greater myocardial thickness and higher incidence of the presence of myocardial sleeves around the CS. These sleeves are absent in the great cardiac vein and the valve of Viussens or the Vein of Marshall demarcates the junction between the CS and the GCV. More distal/"anterior" positions are associated with greater proximity to the circumflex artery with greater risk of circumflex arterial damage. We recommend mitral isthmus ablation at between 3 to 4 o'clock on the mitral valve annulus when viewed in the left anterior oblique projection. These early studies are limited by the lack of correlation between the anatomic obstacles and the likelihood of ablation success.

More recently, clinical studies have sought to examine the impact of these morphologic features on the success of mitral isthmus ablation. Takatsuki et al. found that "high take-off" left inferior pulmonary vein [defined as the lower border of the vein having a more cranial position when compared to the top of the mitral annulus on computed tomography (CT) imaging] was associated with a significantly lower success rate (50% vs 87%) [46]. They suggested that high take-off LIPV may make catheter manipulation more difficult and may result in poor catheter stability. In a recent publication, Yokokawa et al. found that presence of a "pouch" and increased myocardial thickness in the area were associated with failure to achieve mitral isthmus block, although they were not significant in multivariate analysis [34]. Longer mitral isthmus length was also associated with reduced ablation success [47].

Another unique anatomical feature of the mitral isthmus line is the presence of epicardial vascular structures such as the CS and circumflex artery near to the annulus. Blood flow in these structures may act as an epicardial "heat-sink", removing heat from the ablation site by convective cooling, thereby reducing the efficacy of radiofrequency ablation [43]. Hence, there may be a need to perform ablation in the CS itself to target the "protected" epicardial aspect of the myocardium. A previous in-vitro study demonstrated this "heat-sink" effect when flow in the marginal artery prevented the formation of transmural lesions in rabbit ventricular myocardium [48]. There is increasing evidence to support this hypothesis. We showed that a larger CS diameter was significantly associated with the need for CS ablation as well as the total ablation time needed to achieve block [47]. Yokokawa et al. reported a higher prevalence of an interposed circumflex artery between the CS and mitral isthmus in cases of unsuccessful mitral isthmus ablation [34]. In that study, presence of an interposed artery was the only independent predictor of failure to achieve mitral isthmus block. In another recent study, Kurotobi et al. found that the presence of a local coronary artery and CS diameter were independent risk factors for unsuccessful mitral isthmus ablation [49]. The authors advocate pre-ablation imaging with angiography or CT to visualise local coronary artery to help predict the likelihood of achieving mitral isthmus block.

In addition, there are myocardial sleeves around the CS and also the Vein of Marshall which may act as epicardial bridges of connection [50]. Endocardial ablation may result in local block across the atrial myocardium with ongoing conduction through these epicardial structures [51], necessitating high-density endocardial and epicardial mapping (via the coronary sinus and great cardiac vein) to identify insertion points.

## Complications

Unsurprisingly, in view of the extensive ablation (including within the CS) to achieve mitral isthmus block and the close relationship of the circumflex artery and esophagus to the mitral isthmus and CS, cardiac tamponade, circumflex artery damage and atrio-esophageal fistula have been reported in the literature [3,52-543,52-54]. Jais et al. noticed a risk of cardiac tamponade (2 out of 136) in their original study which was attributed to the delivery of more than 50W endocardially using an irrigated tip catheter [3]. He subsequently recommended limiting the maximum power to 42W endocardially. In our centre, we often limit the maximum power to 40-50W at the annular end of the mitral isthmus line (overlying the CS) and we have not noticed an excessive risk of cardiac tamponade. In our experience, 50W is sometimes necessary to achieve mitral isthmus block.

**Figure 5:**
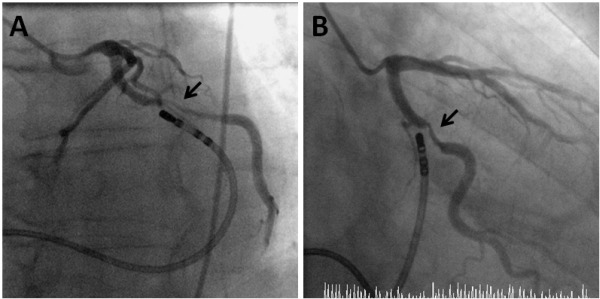
Coronary angiography with the ablation catheter positioned in the coronary sinus (CS) in the left anterior oblique (A) and the right anterior oblique (B) views. The relationship between the CS (represented by the ablation catheter) and the circumflex artery becomes closer at a more "anterior"/distal position of the CS. Arrow indicates spasm of the circumflex artery after ablation at a very "anterior" position.

There is a very close relationship between the circumflex artery and the CS. Hasdemir et al. found that the circumflex artery was <2mm from the CS catheter at the lateral and anterolateral mitral annulus in 24% of patients [55]. This relationship may be even closer at a more distal, "anterior" position (i.e. earlier than 3 o'clock on the mitral annulus) (Figure 5). Wittkampf et. al. postulated that the risk of damage to the circumflex artery is increased with more 'distal' ablation [43]. Takahashi et al. reported the first case of acute circumflex artery occlusion during CS ablation to achieve mitral isthmus block [52]. The incidence was estimated at 1 in 499 patients in their centre. The low incidence may be explained by the arteries being "protected" by the "heat-sink" effect secondary to the blood flow. In one case report, underlying atheromatous disease may have predisposed to occlusion due to reduced protection by impaired blood flow [53]. However, the risk factors for coronary artery damage remained unclear. We reported a high incidence (28%) of asymptomatic circumflex artery "injury" (stenoses ranging from 50%-80%) noticed on coronary angiography after mitral isthmus ablation [56]. These stenoses all resolved with intra-coronary nitrates which led us to postulate that the most likely mechanism was thermally-induced spasm. Ablation within the CS, proximity of the circumflex and CS and a small calibre distal circumflex were found to be risk factors for circumflex artery injury. A pre-procedural coronary angiogram may be helpful to help assess the risk of circumflex artery injury (especially if there was significant circumflex artery disease or if the circumflex artery was located very close to the CS). Yokokawa et al. also suggested that pre-procedural imaging with CT may identify interposed circumflex artery which may deter operator from performing mitral isthmus ablation [34].

The CS is also closely related to the esophagus and hence ablation here may potentially lead to the serious complication of atrio-esophageal fistula. Lemola et al. found that 80% of patients had a mean separation between the CS and esophagus of about 1mm [57]. In our centre, we find the use of esophageal temperature probes helpful in visualizing the oesophagus and alerting us when the temperature rise in the esophagus is excessive.

## Practical approach

While the use of a 8mm-tip non-irrigated catheter and/or non-deflectable sheaths had been described previously, we prefer to use an irrigated-tip catheter via a deflectable sheath in our laboratory for added stability and manoeuvrability. Ablation is also guided by 3-D electroanatomical systems.

**Figure 6:**
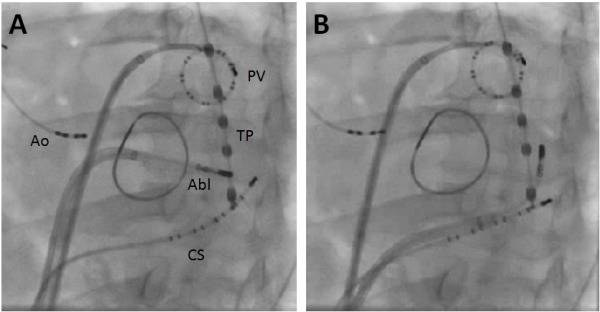
Left anterior oblique fluoroscopic picture showing the catheter positions when performing mitral isthmus ablation from the endocardial surface (A) and epicardially in coronary sinus (B). Quadripolar catheter (Ao) is positioned in the aortic root as reference catheter. Decapolar catheter (CS) is positioned in coronary sinus. Abl refers to ablation catheter. The temperature probe (TP) is positioned in the esophagus. A mitral annuloplasty ring is also seen

A decapolar catheter is positioned in the coronary sinus (CS) so that the distal bipole is just "posterior" to (below) the intended ablation line. We also position the circular mapping catheter used for confirmation of pulmonary vein isolation in the left atrial appendage (LAA) for pacing above the line as the activation pattern in the coronary sinus provides a beat-to-beat assessment of mitral isthmus block during mitral isthmus ablation. The sheath-catheter apparatus is then positioned in the lateral mitral valve annulus (usually between 3 and 4 o'clock in a left anterior oblique view) guided by fluoroscopy (Figure 6A), 3D mapping system and electrograms. Firstly, the sheath is deflected and the catheter is extended out of the sheath into the left ventricle. Then, with gentle clockwise torque of the sheath and gradual withdrawal of the catheter into the sheath, the catheter is positioned with an atrioventricular ratio of 1:2 at the lateral mitral annulus just above the CS distal bipole.

Ablation is then performed either by the "point-by-point" or the "drag" technique. Recommended ablation settings: power: 40-50W at the anterior annular end and 30-40W at the posterior venous end of line; temperature: 43-48ºC; irrigation: 17-30mls/min. Further clockwise torque of the sheath and withdrawal of the catheter will allow the tip of the ablation catheter to be moved posteriorly towards the LIPV. Small adjustments to the deflection of the sheath can be made to raise or lower the ablation catheter to take the shortest route to the LIPV. Ablation is guided by the reduction of local electrogram amplitude. If there is no mitral isthmus block, the catheter is moved along the line or just below the line to look for sharp atrial electrograms which are earlier than the electrogram on the CS distal bipole.

If there are no sharp electrograms which are earlier than CS distal bipole, ablation is performed epicardially in the CS at a site overlying the endocardial line. The sheath-catheter apparatus is withdrawn into the right atrium. The catheter is advanced into the distal CS beyond the CS distal bipole with the sheath positioned at the CS os for support. The ablation catheter is deflected towards the endocardial surface opposite to the lesions delivered endocardially to minimise the risk of circumflex artery damage (Figure 6B). Recommended ablation settings are: power: 25-30W; temperature: 43-48ºC; irrigation: 17-30mls/min.

**Figure 7:**
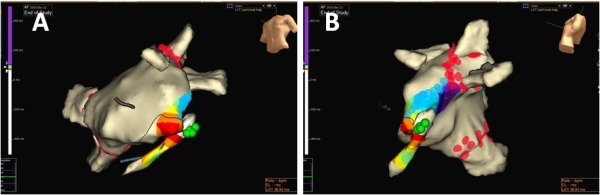
Activation maps in left anterior oblique (A) and left lateral (B) views during pacing from the left atrial appendage showing the breakthrough point in distal coronary sinus (represented by the earliest activation, white colour). Ablation in the coronary sinus (green lesions) subsequently achieved mitral isthmus block. The red lesions represent circumferential pulmonary vein ablation and blue lesions represent endocardial mitral isthmus ablation.

If required, an electroanatomical activation map could be performed just below the mitral isthmus line during LAA pacing to help identify the earliest breakthrough site (Figure 7).

In spite of all these efforts, acute mitral isthmus block may not be achieved in up to about 5-10% of patients, presumably due to local myocardial edema preventing full-thickness necrosis.

## Alternative Isthmus

Previously, an alternative septal/medial left atrial isthmus (from the right inferior pulmonary vein to the mitral annulus) was suggested as an alternative to the lateral mitral isthmus. However, it was found to have a less favourable anatomy, i.e. a longer isthmus and greater percentage of ridges [45]. Wittkampf et al. also found that the myocardial thickness increases at a more medial mitral isthmus position [43]. In a group of patients undergoing surgical cryo-ablation with concomitant valve surgery, bi-directional block was achieved in about two-thirds of lateral isthmus ablation but not achieved in any medial isthmus ablation [30].

Tzeis et al. reported on the safety and feasibility of a modified anterior line extending from the anterior/anterolateral mitral annulus to the orifice of the left superior pulmonary vein, just medial to the LAA [58]. Although it was very effective in terminating perimitral flutters (>95%), bi-directional block was only achieved in 86% of cases. The mean procedure and ablation time were 28.9 mins and 16.6 minutes respectively, which were comparable to those reported for the conventional lateral mitral isthmus. With this technique, there is no need for ablation within the CS which may be desirable in view of the complications associated with CS ablation. Inadvertent isolation of the LAA or delayed activation of the LAA may be potential drawbacks.

Pak et al. have recently taken this further by proposing voltage map-guided left atrial anterior wall ablation [59]. He reported a correlation between low voltage areas on the left atrial anterior wall and the LA-aorta contact area on multislice cardiac magnetic resonance scan. The mean voltage in this area was significantly lower than that at the lateral mitral annulus. Although the mean length of the ablation line at this anterior site was longer than the lateral mitral isthmus, they reported a higher success rate at achieving bi-directional block (albeit only 68% vs 32%). It was also associated with a better clinical outcome after single procedure.

## New techniques

In order to overcome potential obstacles to mitral isthmus ablation and to improve success and ease of ablation, several novel techniques have been suggested. There is increasing evidence to support the "heat-sink" hypothesis (see above). To reduce the "heat-sink" effect, the blood flow in the CS could be temporarily stopped with an occlusion balloon. D'Avila et al. first reported that balloon occlusion of the CS facilitated transmural ablation at the mitral isthmus in an animal study [60]. We performed a randomized study in humans and also found that balloon occlusion of the CS using an air-filled balloon improved mitral isthmus ablation by significantly reducing the need for epicardial CS ablation, as well as reducing mean total and CS ablation times [61]. However, in our study, acute success rate was not significantly improved. Despite the promising results, we cannot recommend this to be performed routinely due to the lack of custom-designed kit for balloon occlusion of the CS.

Another potential barrier to successful mitral isthmus ablation is the lack of stability of the ablation catheter and poor tissue contact. Steerable sheaths could confer the theoretical benefit of improved navigation and stability [62]. Specifically, they could allow for the more parallel orientation of the ablation catheter to the mitral isthmus to improve ablation efficacy, but also allow the ablation catheter to be positioned perpendicularly to "dig" into pouches, which could be possible anatomical barriers to successful mitral isthmus ablation [34]. The use of steerable sheaths had previously been demonstrated to facilitate PVI [63-65] and CTI ablation [66]. A recent randomized study showed that steerable sheaths significantly improve the efficacy of ablation at the mitral isthmus [62]; the acute rate of mitral isthmus block was 97.5%, mean ablation time was 11.8±6.4 mins and the need for CS ablation was only 12.5%. This finding represented a major improvement on previous published results (Table 2).

We have recently reported on the safety and feasibility study on mitral isthmus ablation using a robotic navigation system (Hansen Sensei Robotics). This study enrolled the first 30 patients who had mitral isthmus ablation and the results included our learning curve. Overall, we achieved an acute success rate of 97%, the need for CS ablation was 53% and mean ablation time was 14±7 min [67].

Point-by-point ablation is time-consuming and challenging and may lead to the creation of non-contiguous lesions which may be pro-arrhythmic. Circular multielectrode ablation catheters using duty-cycled bipolar and unipolar radiofrequency (RF) energy have been demonstrated to be effective in achieving pulmonary vein isolation [68]. Using the same technology, a novel hexapolar linear multielectrode catheter has been developed and has the potential to create contiguous lesions with less radiofrequency applications. In addition to the ability to deliver radiofrequency energy simultaneously from all poles, it also allows for simultaneous mapping for the entire length and can potentially aid in the prompt identification of conduction gaps [69]. While it may be promising for CTI ablation [70], the initial experience for mitral isthmus ablation has been disappointing with an acute success of only 29% [71]. Further bigger multi-centre studies are needed to fully assess the impact of this technology on mitral isthmus ablation.

In view of possible circumflex artery and oesophageal damage, our group had explored the possibility of using cryo-ablation (delivered in a point-by-point fashion using Freezor Max with an 8 mm ablation tip). While cryo-ablation was safe and effective, it is currently too time consuming for it to be a practical alternative.

## Conclusion

While the acute success rate of mitral isthmus ablation is continuing to improve due to better technology and tools, recurrence of conduction still occurs in many cases and may pre-dispose patients to clinical tachycardia. The challenge remains to achieve durable mitral isthmus block. The benefits of adjunctive mitral isthmus ablation also needs to be clarified by well-designed randomised controlled trials.

## Figures and Tables

**Table 1:**
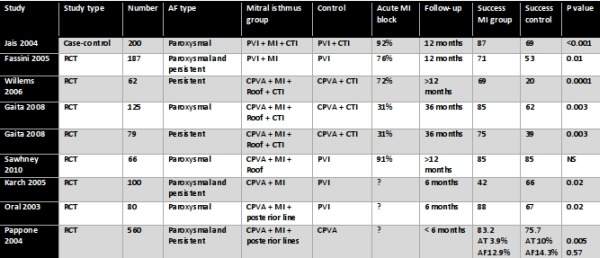
Studies reporting on the efficacy of mitral isthmus ablation as an adjunct to pulmonary vein isolation

CPVA = circumferential pulmonary vein ablation; CTI = cavotricuspid isthmus; MI = mitral isthmus; PVI = pulmonary vein isolation; RCT = randomised controlled trial

**Table 2:**
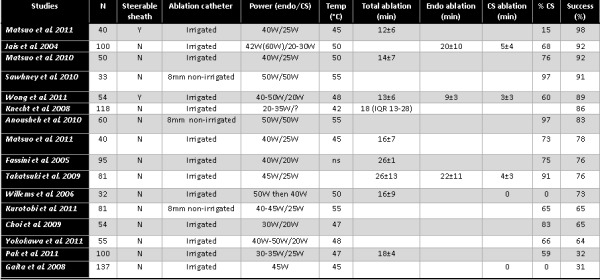
Studies reporting on acute success rate of mitral isthmus ablation

CS = coronary sinus; Endo = endocardium
